# Characterization of a MHYT domain-coupled transcriptional regulator that responds to carbon monoxide

**DOI:** 10.1093/nar/gkae575

**Published:** 2024-07-05

**Authors:** Gonzalo Durante-Rodríguez, Sofía de Francisco-Polanco, José Luis García, Eduardo Díaz

**Affiliations:** Department of Microbial and Plant Biotechnology, Centro de Investigaciones Biológicas Margarita Salas-CSIC, Calle Ramiro de Maeztu, 9, 28040 Madrid. Spain; Department of Microbial and Plant Biotechnology, Centro de Investigaciones Biológicas Margarita Salas-CSIC, Calle Ramiro de Maeztu, 9, 28040 Madrid. Spain; Department of Microbial and Plant Biotechnology, Centro de Investigaciones Biológicas Margarita Salas-CSIC, Calle Ramiro de Maeztu, 9, 28040 Madrid. Spain; Department of Microbial and Plant Biotechnology, Centro de Investigaciones Biológicas Margarita Salas-CSIC, Calle Ramiro de Maeztu, 9, 28040 Madrid. Spain

## Abstract

The MHYT domain, identified over two decades ago for its potential to detect diatomic gases like CO, O_2_ or NO, has awaited experimental validation as a protein sensory domain. Here, we characterize the MHYT domain-containing transcriptional regulator CoxC, which governs the expression of the *cox* genes responsible for aerobic CO oxidation in the carboxidotrophic bacterium *Afipia carboxidovorans* OM5. The C-terminal LytTR-type DNA-binding domain of CoxC binds to an operator region consisting of three direct repeats sequences overlapping the –35 box at the target *P_coxB_* promoter, which is consistent with the role of CoxC as a specific transcriptional repressor of the *cox* genes. Notably, the N-terminal transmembrane MHYT domain endows CoxC with the ability to sense CO as an effector molecule, as demonstrated by the relief of CoxC-mediated repression and binding to the *P_coxB_* promoter upon CO exposure. Furthermore, copper serves as the essential divalent cation for the interaction of CO with CoxC, thereby confirming previous hypothesis regarding the role of copper in the gas-sensing mechanism of MHYT domains. CoxC represents the prototype of a novel subfamily of single-component LytTR transcriptional regulators, characterized by the fusion of a DNA-binding domain with a membrane-bound MHYT sensor domain.

## Graphical Abstract

**Graphical Abstract:**
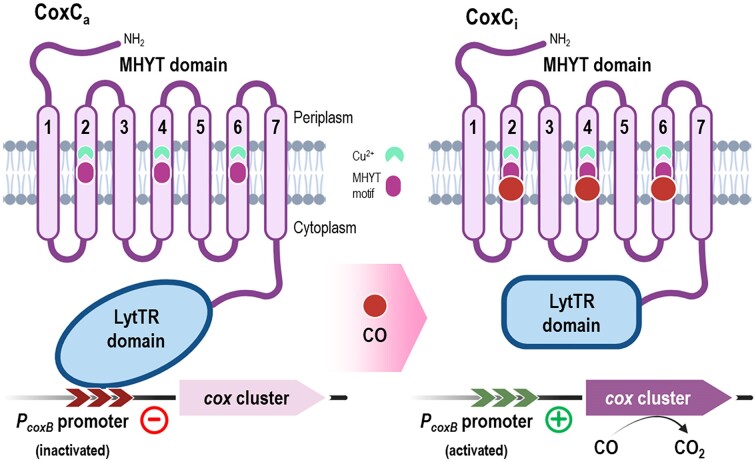

## Introduction

MHYT is a protein domain (Pfam PF03707) (1) approximately 200 residues in length, which contains seven predicted transmembrane segments connected via cytoplasmic and periplasmic loops rich in charged amino acids (aa). Three of these transmembrane segments contain a consensus sequence motif (MHYT motif) with highly conserved Met, His and Tyr residues located near the periplasmic side of the cytoplasmic membrane. These conserved residues are predicted to coordinate copper ions, enabling the sensing of diatomic gases such as oxygen (O_2_), carbon monoxide (CO), or nitric oxide (NO), and thus, suggesting that MHYT acts as a sensory protein domain (2). MHYT can be found either as a separate single-domain protein or fused to signal transduction domains such as HK (histidine kinase), GGDEF (c-di-GMP diguanylate cyclase), EAL (c-di-GMP phosphodiesterase), PAS (Per-Arnt-Sim), and DNA-binding domains, further suggesting a sensory function for the MHYT domain (1–3). Although proteins containing the MHYT domain have been identified in protein databases in >2000 different species, including proteobacteria, cyanobacteria, gram-positive bacteria, and several fungi (1), only a few of these proteins associated with processes controlled by c-di-GMP levels have been thoroughly investigated. For instance, *Pseudomonas aeruginosa* contains two membrane-bound proteins, i.e. NbdA and MucR, with identical MHYT-GGDEF-EAL domain organization. NO-induced biofilm dispersion is mediated by the MHYT domain-coupled c-di-GMP phosphodiesterase (the product of the *nbdA* gene), whose expression is activated after NO treatment, enhancing NbdA synthesis and leading to reduced levels of c-di-GMP in *P. aeruginosa* (4). Conversely, nitrate-dependent regulation of alginate production is mediated by the membrane-anchored MucR protein, with its MHYT domain likely involved in the nitrate response through NO sensing (5). The protein DgcW (formerly YkoW) from *Bacillus subtilis* displays a similar domain architecture to MucR and NbdA and has been shown to possess diguanylate cyclase activity (6). More recently, the CdgB protein from *Azospirillum baldaniorum*, exhibiting a MHYT-PAS-GGDEF-EAL domain organization, was reported to be a bifunctional enzyme with both c-di-GMP cyclase and phosphodiesterase activity, with its MHYT domain proposed to modulate both activities by sensing NO during the switch between sessile or motile lifestyles (7). However, despite the description of the MHYT domain and its predicted function as a sensor of diatomic gases over 20 years ago, experimental proof of this assumption has not yet been reported.

**Figure 1.**
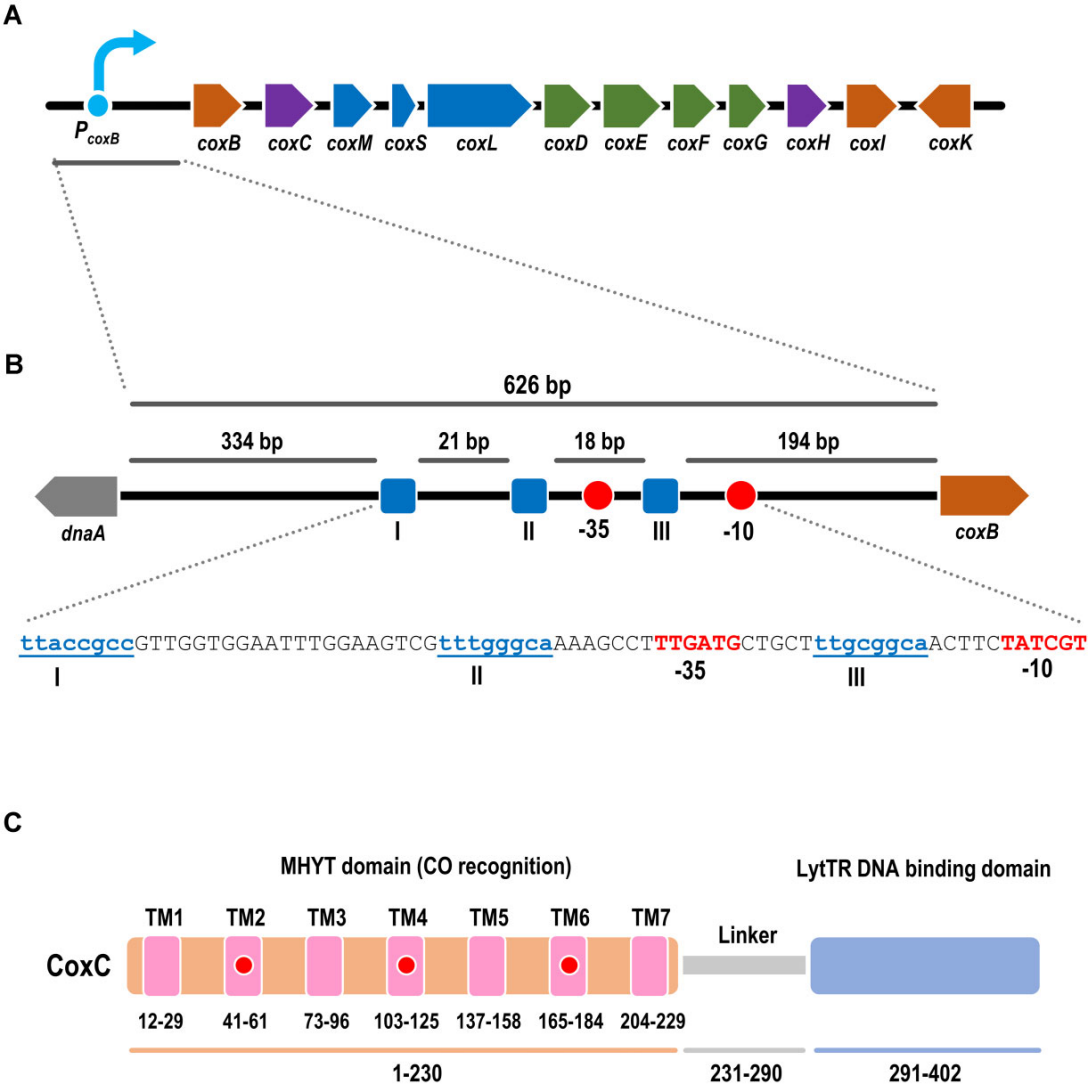
The *cox* cluster of *A. carboxidovorans* OM5 and its regulatory elements. **(A)** Scheme of the *cox* cluster. The genes *coxMSL* encode the aerobic CO dehydrogenase (blue), *coxDEFG* encode proteins involved in the post-translational biosynthesis of the [CuSMoO_2_] cluster of CO-dehydrogenase (green)*, coxC* and *coxH* encode putative transcriptional regulators (violet), *coxB* and *coxK* encode putative transmembrane proteins of unknown function, and *coxI* encodes a cytoplasmic protein of unknown function (brown). The *P_coxB_* promoter is respresented in blue. **(B)** Scheme of *P_coxB_* promoter. The intergenic region between *dnaA* and *coxB* genes (626 bp) is represented. The blue squares (I, II and III) show the directed repeat sequences, and red circles show the putative –10 and –35 boxes of the *P_coxB_* promoter. An expanded view of the sequence of the three direct repeats (blue), the spacers, and the predicted –35 and –10 boxes of σ^70^-dependent promoters (red), is shown at the bottom. **(C)** Scheme of the CoxC primary structure. N-terminal region with MHYT domain (aa residues 1–230) is depicted in orange: transmembrane segments TM1 to TM7 are indicated in pink, and the three MHYT motifs (aa residues 57–60, 118–121 and 180–183) predicted to interact with CO are represented with red circles. C-terminal region with LytTR DNA-binding domain (aa residues 291–402) is shown in blue. A linker region connecting the N- and C-terminus is shown in grey.

Some proteins (371 proteins in InterPro IPR012073 database, mostly encoded in α-proteobacterial genomes) contain a MHYT domain fused to a DNA-binding domain belonging to the LytTR family (8). This protein architecture is found in the CoxC and CoxH proteins encoded in the *cox* cluster of the α-proteobacterium *Afipia carboxidovorans* OM5 (formerly *Oligotropha carboxidovorans* OM5) (9,10). The *cox* cluster comprises 12 genes encoding the proteins involved in the aerobic metabolism of CO: *coxMSL* encode the CO dehydrogenase enzyme that generates CO_2_ and energy from CO; *coxDEFG* encode proteins mainly involved in the posttranslational synthesis and maturation of the bimetallic [CuSMoO_2_]-center in the active site of CO dehydrogenase; *coxI* encodes a cytoplasmic protein; and *coxB*, *coxC*, *coxH* and *coxK* genes encode putative transmembrane proteins (Figure 1A) (9,11–13). Since CoxC and CoxH contain a DNA-binding domain, they have been proposed as the putative transcriptional regulators of the CO-induced *cox* genes (8,14), but this hypothesis has not been proven yet.

In this work, we characterize for the first time a MHYT domain-containing transcriptional regulator, the CoxC protein, and its cognate promoter (*P_coxB_*), which control the expression of the *cox* genes in *A. carboxidovorans*. The interaction between the membrane-bound CoxC regulator and the *P_coxB_* promoter is specifically alleviated in the presence of CO, thus behaving as an effector molecule. Interestingly, this interaction is reversible and requires the presence of copper ions. Overall, this work experimentally demonstrates the long-standing assumption that the widespread MHYT domain senses diatomic gases and reveals that CoxC constitutes the prototype of a new subfamily of membrane-bound single-component LytTR transcriptional regulators.

## Materials and methods

### Bacterial strains, plasmids and growth conditions

The *Escherichia coli* and *A. carboxidovorans* strains, along with the plasmids utilized in this study, are outlined in Supplementary Table S1. *E. coli* cells were cultivated aerobically at 37ºC with continuous agitation in lysogeny broth (LB) medium (15) or in M63 minimal medium (16), supplemented with the necessary nutrients, and 20 mM glycerol as the carbon source. Where appropriate, antibiotics and isopropyl-1-thio-ß-d-galactopyranoside (IPTG) were added at the following concentrations: ampicillin (Ap, 150 μg/ml), kanamycin (Km, 50 μg/ml), gentamicin (Gm, 10 μg/ml), and IPTG (1 mM). *A. carboxidovorans* cells were cultivated aerobically at 30ºC with continuous agitation in M63 minimal medium (16), supplemented with the necessary nutrients, and 0.2% pyruvate as the carbon source. For gas-induced cultures, the cells were cultivated in stoppered serum vials with a headspace of 120 ml containing 10 ml of culture medium, with 2% (vol/vol; *E. coli*) filtered gas CO or CO_2_ (provided by Air Liquide).

### Molecular biology techniques

Recombinant DNA techniques were conducted following published methods (15,16). Plasmid DNA was isolated using the High Pure Plasmid Isolation Kit (Roche Applied Science). DNA fragments were purified using Gene Clean Turbo (Q-BIOgene). Oligonucleotides were supplied by Sigma. All cloned inserts and DNA fragments were verified by DNA sequencing performed with an ABI Prism 377 automated DNA sequencer (Applied Biosystems). Transformation of *E. coli* was performed either using the RbCl method or by electroporation (Gene Pulser, Bio-Rad) (16). Proteins were analyzed using an SDS-PAGE system (15) with a 15% acrylamide/bisacrylamide (37.5:1) gel cast in a Mini-PROTEAN 3 Cell (Bio-Rad), following standard protocols. Proteins were resuspended in a denaturing buffer containing 2% sodium dodecyl sulfate (SDS), 5% glycerol, 60 mM Tris–HCl pH 6.8, 1% β-mercaptoethanol and 0.005% bromophenol blue, and boiled for 10 min prior to loading. Gels were subjected to Coomassie staining as described previously (15). Protein concentration in cell fractions was determined using the method of Bradford (17), employing serum albumin as the standard.

### Construction of pIZ2-CoxC, pIZ2-C-CoxC, pQE32-His_6_C-CoxC, pSEVA225-PcoxB and pSEVA225-PcoxB1 plasmids

To construct plasmid pIZ2-CoxC, a 1209 bp DNA fragment containing the *coxC* gene was PCR amplified from *A. carboxidovorans* OM5 by using oligonucleotides 5CoxCEco2 (5′-GCGAATTCGCTTAGGAGGAAAAACATATGCCCATCACACACGATCCGATG-3′, with engineered *Eco*RI underlined) and 3CoxCHind (5′-GGGAAGCTTTCAACTGCCTTTCGTCCCCAAATAC-3′, with engineered *Hin*dIII underlined). For plasmid pIZ2-C-CoxC, expressing the DNA binding domain of CoxC, a 489 bp DNA fragment containing the *C-coxC* gene was PCR amplified from *A. carboxidovorans* OM5 using oligonucleotides 5C-CoxCEco (5′-GCGAATTC ATTTTGCCCGAATCGAGTGCGTCTAC -3′, with engineered *Eco*RI underlined) and 3CoxCHind (as above). Subsequently, the PCR amplified products were cloned into the *Eco*RI/*Hin*dIII double-digested pIZ2 vector, resulting in plasmids pIZ2-CoxC and pIZ2-C-CoxC, respectively (Supplementary Table S1). For the recombinant plasmid pQE32-His_6_C-CoxC overexpressing the DNA binding domain of CoxC, PCR amplification was conducted from *A. carboxidovorans* OM5 using oligonucleotides 5C-CoxCBam (5′-CGGGATCCTTATTTTGCCCGAATCGAGTGCGTCTAC-3′, with engineered *Bam*HI underlined) and 3CoxCHind (as above). The resulting 489 bp PCR amplified product was digested with *Bam*HI and *Hin*dIII restriction enzymes, then ligated to the *Bam*HI/*Hin*dIII double-digested pQE32 His_6_-tag vector, yielding plasmid pQE32-His_6_C-CoxC (Supplementary Table S1). This recombinant plasmid expresses, under control of the *T5* promoter and two *lac* operator boxes, the His_6_C-CoxC protein that carries a 12-amino acid sequence (MRGSHHHHHHGIL) fused to the N-terminus of the LytTR DNA-binding domain (aa residues 240 to 402). To construct the recombinant plasmids pSEVA225-PcoxB and pSEVA225-PcoxB1, a 282 bp DNA fragments containing the *P_coxB_* or *P_coxB1_* promoter were PCR amplified from *A. carboxidovorans* OM5 using oligonucleotides 5PcoxBEco (5′- GCGAATTCCAGTTCATGCGTATGTTACCGCCGTTG-3′, with engineered *Eco*RI underlined) for *P_coxB_*, or 5PcoxB1Eco (5′-GCTCGAATTCCAGTTCATGCGTATG**GG**ACC**C**CCGTTG-3′, engineered *Eco*RI site underlined, substitutions in repeat 1 are in bold) for *P_coxB1_*, and 3PcoxBBam (5′-CGGGATCCCAATATCGTCCGCGAAAATGCGGTC-3′, with engineered *Bam*HI underlined). Subsequently, the PCR amplified products were digested with *Eco*RI and *Bam*HI restriction enzymes, then ligated to the *Eco*RI/*Bam*HI double-digested pSEVA225 vector, resulting in plasmid pSEVA225-PcoxB and pSEVA225-PcoxB1 (Supplementary Table S1).

### Overproduction and purification of His_6_C-CoxC protein

The His_6_C-CoxC protein was overproduced in *E. coli* M15 strain carrying plasmid pQE32-His_6_C-CoxC, along with the pREP4 plasmid that produces the LacI repressor to strictly regulate gene expression from pQE32 derivatives in the presence of IPTG. *E. coli* M15 (pREP4, pQE32-His_6_C-CoxC) cells were cultivated at 37°C in 200 ml of LB medium containing ampicillin and kanamycin until reaching mid-exponential growth phase. Overexpression of the His_6_-tagged protein was induced for 24 h by adding 1 mM IPTG. Cells were then harvested at 4°C, resuspended in 10 ml of lysis buffer (50 mM NaH_2_PO_4_, 300 mM NaCl, 20 mM imidazole, pH 8.0), and disrupted by passage through a French press (Aminco Corp.) operated at a pressure of 20000 p.s.i. The resulting cell lysate was centrifuged at 26000 × *g* for 30 min at 4°C. The clear supernatant fluid was carefully decanted and applied to nickel-nitrilotriacetic (Ni-NTA) acid-agarose columns (Qiagen). Subsequently, the columns were washed at 4°C with 50 volumes of washing buffer (50 mM NaH_2_PO_4_, 300 mM NaCl, 20 mM imidazole, pH 8.0), and the His_6_C-CoxC protein was eluted using elution buffer (50 mM NaH_2_PO_4_, 300 mM NaCl, 500 mM imidazole, pH 8.0). The purified protein was then dialyzed at 4°C in dialysis buffer (20 mM NaH_2_PO_4_, 100 mM NaCl, 10% glycerol, 2 mM β-mercaptoethanol, pH 8.0) and stored at –20°C.

### β-Galactosidase assays

The β-galactosidase activities from the *P_coxB_::lacZ* reporter fusion were measured with permeabilized cells when cultures reached mid-exponential phase as described previously (16).

### CoxC-enriched membrane fractions

CoxC was produced in an *E. coli* DH10B strain carrying plasmid pIZ2-CoxC. *E. coli* DH10B (pIZ2-CoxC) cells were cultured at 37°C in 50 ml of LB medium supplemented with gentamicin, with 1 mM IPTG added to induce expression of the *coxC* gene. After cultivation, cells were harvested at 4°C, resuspended in 5 ml of lysis buffer (50 mM NaH_2_PO_4_, 300 mM NaCl, 20 mM imidazole, pH 8.0), and disrupted by passage through a French press (Aminco Corp.) operated at a pressure of 20000 p.s.i. Then, 1 ml of the cell lysate was centrifuged at 26000 × *g* for 30 min at 4°C. The soluble fraction was discarded and the membrane (sedimented) fraction was resuspended in 1 ml of membrane buffer (25% sucrose, 20 mM Tris, pH 8, and 0.5 mM phenylmethylsulfonyl fluoride PMSF) (18). Control membrane fractions (lacking CoxC) were obtained using the same procedure but with *E. coli* DH10B (pIZ2) cells.

### Electrophoretic mobility shift assays (EMSA)

The 282 bp PcoxB probe and the different mutant probes PcoxB1, PcoxB2 and PcoxB3, each with substitutions at the CoxC-binding sites, were PCR-amplified using the pSEVA225-PcoxB plasmid as template and specific primer pairs. The forward oligonucleotides used were 5PcoxBEco (previously mentioned), 5PcoxB1Eco (previously mentioned), 5PcoxB2Eco (5′-GCTCGAATTCCAGTTCATGCGTATG**GG**ACC**C**CCGTTGGTGGAATTTGGAAGTCG**GG**TGG**C**CAAAAG-3′, engineered *Eco*RI underlined, substitutions in bold), or 5PcoxB3Eco (5′-GCGAATTCCAGTTCATGCGTATG**GG**ACC**C**CCGTTGGTGGAATTTGGAAGTCG**GG**TGG**C**CAAAAGCCTTTGATGCTGCT**GG**GCG**C**CAACTTCTATCGTG-3′, engineered *Eco*RI underlined, substitutions in bold), and the reverse oligonucleotide used was 3PcoxBBam (previously mentioned). The resulting DNA products were digested with *Eco*RI restriction enzyme and 3′ end-labeled by filling in the overhanging *Eco*RI-digested ends with [α-^32^P]-dATP (6000 Ci/mmol; PerkinElmer Life Sciences) and the Klenow fragment of *E. coli* DNA polymerase I, as described previously (15). Electrophoretic mobility shift assays (EMSA) were conducted in TRRG buffer (20 mM Tris–HCl pH 7.5, 50 mM KCl, 2 mM β-mercaptoethanol, 10% v/v glycerol) containing 2 ng of end-labeled PcoxB, PcoxB1, PcoxB2 or PcoxB3 probes, 250 μg/ml BSA, 50 μg/ml unspecific salmon sperm DNA, 1 mM DTT, 1 mM dithionite, and increasing amounts of purified His_6_C-CoxC or CoxC-enriched membrane fractions in a final volume of 10 μl. Samples were incubated for 20 min at 25ºC, then fractionated by electrophoresis in a 5% non-denaturing polyacrylamide gel buffered with 0.5X TBE (45 mM Tris borate, 1 mM EDTA) (15). The gels were subsequently dried onto Whatman 3MM paper and exposed to Hyperfilm MP (Amersham Biosciences) accompanied by amplifier screens (Cronex Lightning Plus, DuPont).

Where indicated, the CoxC-enriched membrane fractions or purified His_6_C-CoxC protein were subjected to treatment with CO, CO_2_ or air (O_2_), by applying these gases with a needle from a gas cylinder (Air Liquid) for 15 seconds to a 50 μl volume sample in an Eppendorf tube before addition to EMSA. Additionally, 0.2 mM EDTA (ethylenediaminetetraacetic acid) was added to the membrane buffer used to resuspend the CoxC-enriched membrane fractions when necessary. Moreover, when indicated, 0.2 mM CuCl_2_, 2 mM ZnCl_2_, 2 mM NiCl_2_ or 2 mM CoCl_2_ were added to the 50 μl volume sample prior to the application of the corresponding CO gas.

### Sequence data and phylogenetic analyses

For bioinformatic inspection of genes and regulatory regions of interest, we utilized the BioEdit Sequence Alignment Editor (19) and the ApE-A plasmid Editor v.1.17 (Copyright^©^ 2003–2008, M. Wayne David—https://jorgensen.biology.utah.edu/wayned/ape). The BLAST platform (20) was employed to investigate sequence similarity and identity. Amino acid sequences were compared with those in databases using the TBLASTN algorithm on the NCBI server (http://blast.ncbi.nlm.nih.gov/Blast.cgi). The predicted membrane topology of the CoxC protein was visualized with the Protter software (21). Nucleotide and protein alignments were conducted using the ALIGN (22) and CLUSTALW (23) programs, respectively, in the BioEdit editor. The evolutionary history and phylogenetic tree of the CoxC protein were inferred using the Maximum Likelihood method and JTT matrix-based model (24). Evolutionary analyses were performed with the Molecular Evolutionary Genetics Analysis (MEGA11) software (25).

## Results

*coxC* encodes a transcriptional regulator of the *P_coxB_* promoter from *A. carboxidovorans* OM5

The CoxC protein, consisting of 402 amino acids (UniProt: Q9KX27), encoded within the *cox* cluster of *A. carboxidovorans* OM5, exhibits a predicted two-domain structure. It comprises a C-terminal LytTR DNA-binding domain (spanning from aa residues 291 to 402) fused to an N-terminal MHYT domain (spanning from aa residues 1 to 230). The MHYT domain contains three MHYT motifs situated at the termini of the predicted transmembrane segments TM2 (aa positions 41–61), TM4 (aa positions 103–125), and TM6 (aa positions 165–184) (Figure 1C), and predicted to located at the outer face of the cytoplasmic membrane (Supplementary Figure S1) (2,8). These domains are linked by a region (aa residues 231 to 290) predicted to have a disordered structure. Consequently, CoxC is anticipated to function as a transcriptional regulator of the *cox* cluster (8).

The LytTR C-terminal domain of CoxC exhibits moderate amino acid sequence identity (30.4%) with the LytTR domain of the RcoM1 transcriptional regulator, which controls the expression of *cox* genes involved in aerobic CO metabolism in *Paraburkholderia xenovorans* LB400 (26,27). The RcoM1 DNA-binding motif consists of a triplet of direct repeats (consensus sequence TTCNNGCA) evenly spaced at 12–13 bp intervals (28), resembling the pattern of LytTR domain DNA-binding sites (8). Exploration of similar DNA-binding sites within the *cox* cluster of *A. carboxidovorans* identified a putative LytTR operator in the promoter region of the first gene in the cluster (Figure 1B), suggesting that the *coxB* promoter (*P_coxB_*) could be the target site of the putative CoxC regulator.

**Figure 2.**
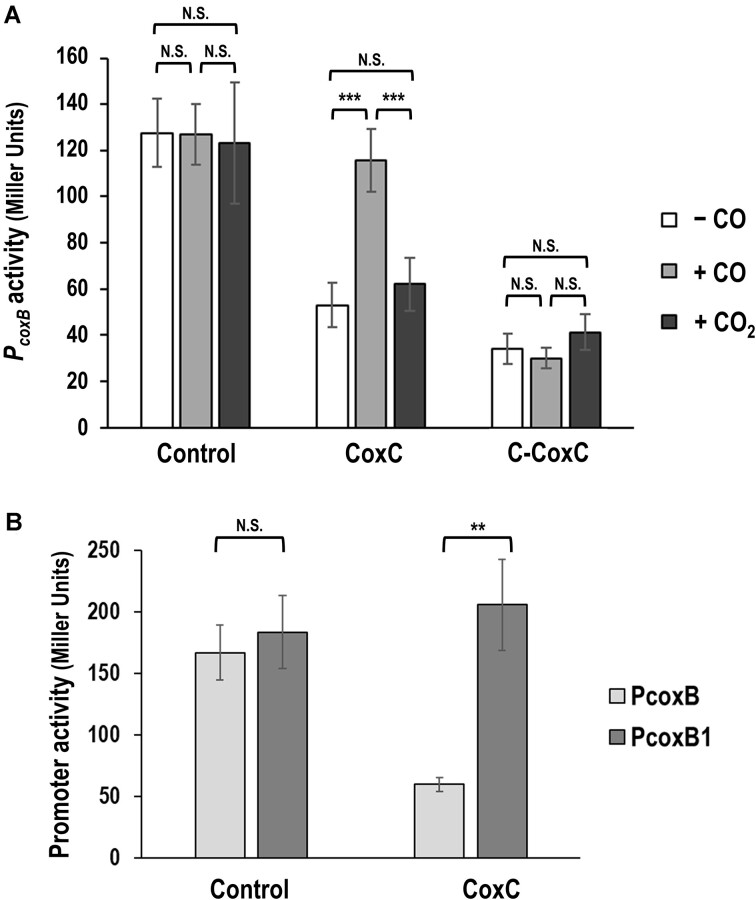
Role of CoxC protein in regulating the *P_coxB_* and *P_coxB1_* promoters. **(A)** Role of CoxC regulating the *P_coxB_* promoter. *E. coli* DH10B (pSEVA225-PcoxB, pIZ2-CoxC) and DH10B (pSEVA225-PcoxB, pIZ2-C-CoxC) cells containing a *P_coxB_::lacZ* fusion and expressing CoxC (CoxC) or C-CoxC (C-CoxC, truncated derivative of CoxC which lacks the MHYT domain), respectively, alongside *E. coli* DH10B (pSEVA225-PcoxB, pIZ2) cells used as control (Control), were cultivated until mid-exponential phase in M63 medium with glycerol as carbon source, 1 mM IPTG, and either lacking CO (– CO) or containing 2% CO (+ CO) or 2% CO_2_ (+ CO_2_). β-galactosidase activity is expressed in Miller units. **(B)** Role of CoxC regulating the *P_coxB1_* promoter. *E. coli* DH10B (pSEVA225-PcoxB, pIZ2-CoxC) and DH10B (pSEVA225-PcoxB1, pIZ2-CoxC) cells expressing *coxC* (CoxC) and containing a *P_coxB_::lacZ* (PcoxB) and *P_coxB1_::lacZ* (PcoxB1) transcriptional fusions, respectively, alongside *E. coli* DH10B (pSEVA225-PcoxB, pIZ2) and DH10B (pSEVA225-PcoxB1, pIZ2) cells used as control (Control), were cultivated until mid-exponential phase in M63 medium with glycerol as carbon source and 1 mM IPTG. β-galactosidase activity is expressed in Miller units. Values are the mean of three independent experiments, with error bars indicating standard deviations. Statistical significance was assessed using the unpaired Student′s *t-*test (two-tailed) between each pair of samples. N.S., not significant differences (*P*> 0.05); asterisks indicate significant differences: ***P< 0.001; **P< 0.01.

To confirm whether CoxC regulates the activity of the *P_coxB_* promoter, a *P_coxB_::lacZ* transcriptional fusion was generated in plasmid pSEVA225-PcoxB (Supplementary Table S1). Additionally, the *coxC* gene was cloned under the control of the IPTG-inducible LacI^q^/*P_tac_* regulatory couple in plasmid pIZ2-CoxC (Supplementary Table S1). β-galactosidase assays conducted in *E. coli* DH10B (pSEVA225-PcoxB, pIZ2-CoxC) and *E. coli* DH10B (pSEVA225-PcoxB, pIZ2) cells revealed that *P_coxB_* was a functional promoter whose activity was inhibited in the presence of CoxC protein (Figure 2A). Thus, these findings demonstrate that CoxC acts as a transcriptional repressor of *P_coxB_*.

### Carbon monoxide (CO) alleviates the CoxC-mediated inhibition of the *P_coxB_* promoter

Previous studies have demonstrated that the expression of *cox* genes is specifically induced in *A. carboxidovorans* cells under chemolithoautotrophic conditions in the presence of CO as the carbon and energy source (9). Thus, we proposed that the CoxC-mediated repression of *P_coxB_* could be relieved when recombinant *E. coli* cells carrying the *P_coxB_::lacZ* fusion and the *coxC* gene were cultivated in the presence of CO. To substantiate this hypothesis, *E. coli* DH10B cells containing plasmids pSEVA225-PcoxB and pIZ2-CoxC were cultured in the absence or presence of 2% CO (a concentration that we have shown it does not cause significant toxicity to *E. coli*), and the activity of the *P_coxB_* promoter was assessed through β-galactosidase assays. Interestingly, CO effectively alleviated CoxC-mediated repression of the *P_coxB_* promoter, leading to a substantial increase in β-galactosidase activity, comparable to that observed in the control strain *E. coli* DH10B (pSEVA225-PcoxB, pIZ2), which carries the *P_coxB_*::lacZ fusion but lacks the CoxC repressor (Figure 2A). Furthermore, when CO was substituted with CO_2_, a non-diatomic gas unlikely to be recognized by MHYT domains, there was no impact on the inhibition of the *P_coxB_* promoter by CoxC (Figure 2A). These findings strongly support the notion that CoxC serves as a transcriptional repressor modulating the activity of the *P_coxB_* promoter in response to CO as an effector molecule.

### Role of CoxC domains

As previously mentioned, the primary structure of CoxC suggests a distinct two-domain architecture (Figure 1C). To verify this domain arrangement and assess whether the C-terminus of CoxC (comprising the LytTR DNA-binding domain) acts as a functional repressor, a truncated version of CoxC spanning from aa 240 to 402 was engineered within plasmid pIZ2-C-CoxC (Supplementary Table S1). This truncated derivative of CoxC, lacking the MHYT domain and denoted as C-CoxC, effectively suppressed the activity of *P_coxB_* in *E. coli* DH10B (pSEVA225-PcoxB, pIZ2-C-CoxC) (Figure 2A), confirming the functionality of the C-terminal LytTR domain in recognizing its target promoter.

Conversely, our observations indicate that the addition of CO did not alleviate the repression of *P_coxB_* caused by C-CoxC (Figure 2A), strongly suggesting that the N-terminal MHYT domain of CoxC is responsible for recognizing and binding to CO.

### The LytTR domain of CoxC binds to a three direct repeats operator sequence

**Figure 3.**
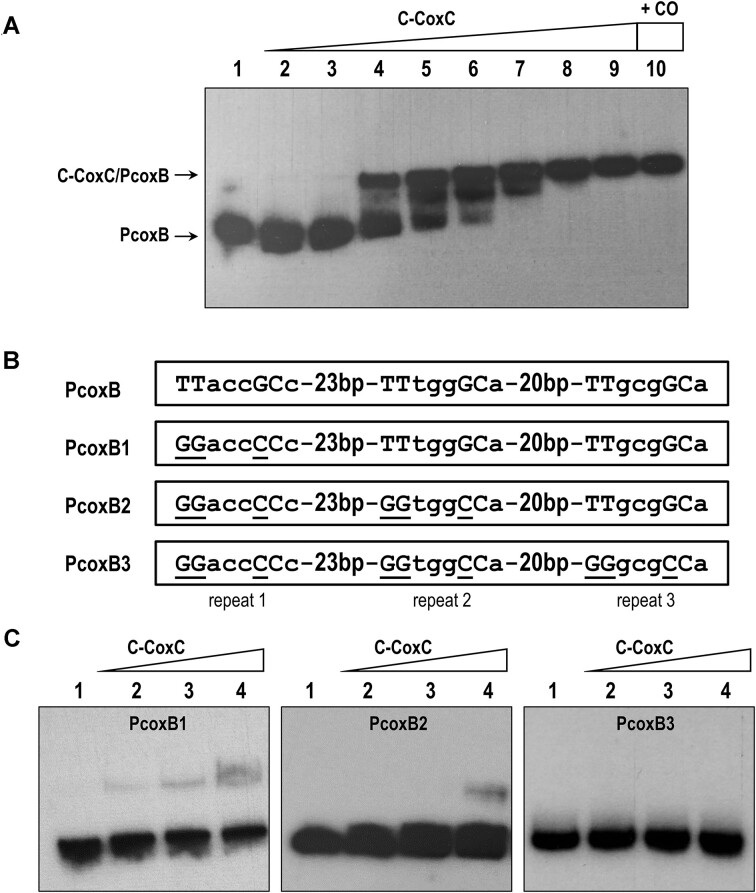
Interaction of purified C-CoxC protein with wild-type and mutant *P_coxB_* promoters. **(A)** EMSA with the wild-type PcoxB probe. Lane 1 shows the free PcoxB probe. Lanes 2 to 9 show EMSA of PcoxB containing 1, 2, 5, 25, 50, 100, 250 and 500 nM of purified His_6_-C-CoxC protein, respectively. Lane 10, shows an EMSA of PcoxB probe with 500 nM His_6_-C-CoxC protein treated with CO. The PcoxB probe and the C-CoxC/PcoxB complex are indicated by arrows. **(B)** Nucleotide sequences of the three direct repeats of wild-type PcoxB probe, as well as those of mutant probes PcoxB1 (repeat 1 mutated), PcoxB2 (repeat 1 and 2 mutated), and PcoxB3 (repeats 1, 2 and 3 mutated). The substituted nucleotides are underlined. **(C)** EMSA with mutant PcoxB probes. Lanes 1, show the free PcoxB1, PcoxB2 and PcoxB3 probes; lanes 2–4, show EMSA of the three probes containing 50, 100 and 250 nM of purified His_6_-C-CoxC protein, respectively.

To further investigate the interaction between C-CoxC and *P_coxB_*, we purified the soluble C-CoxC protein. For the overproduction of the C-CoxC protein with a His_6_-tag at its N-terminal end, the *C-coxC* gene was cloned into the pQE32 vector under the control of the *P_T5_* promoter, resulting in plasmid pQE32-His6C-CoxC (Supplementary Table S1). The His6C-CoxC protein was then overproduced and purified using Ni-NTA columns via affinity chromatography. To validate *in vitro* the interaction of the purified C-CoxC protein with the PcoxB DNA fragment (encompassing 282 bp upstream from the ATG codon of the *coxB* gene), we performed EMSA. The C-CoxC protein effectively slowed the migration of the PcoxB probe in a concentration-dependent manner, exhibiting an apparent K_D_ of approximately 25 nM (Figure 3A). To identify the operator region of CoxC within the *P_coxB_* promoter, we designed three mutant promoter fragments by substituting one (PcoxB1), two (PcoxB2), or three (PcoxB3) TTNNNGCN direct-repeat motifs (Figures 1B and 3B). As previously mentioned, these direct-repeat motifs are proposed to serve as the DNA-binding sites of CoxC, as they are conserved in the operator region of the LytTR transcriptional regulator RcoM1 from *P. xenovorans* LB400 (27,28). EMSA conducted with the C-CoxC protein and the *P_coxB_* mutant promoters revealed very weak binding to the PcoxB1 and PcoxB2 probes, and no binding to the PcoxB3 probe (Figure 3C). Moreover, the *P_coxB1_* promoter, which contains only a single mutated direct-repeat motif, lost the ability to be inhibited by the CoxC protein when tested *in vivo* in *E. coli* cells (Figure 2B). In summary, these findings suggest that the LytTR domain of CoxC specifically interacts with the *P_coxB_* promoter by binding to three conserved direct-repeat motifs spaced by two-helical-turn intervals (18–21 bp) (Figure 1B). Furthermore, the location of the CoxC operator region overlapping the predicted -35 sequence of a σ^70^-dependent promoter (Figure 1B) is consistent with the observation that CoxC functions as a repressor of *P_coxB_* (Figure 2A). The CoxC-mediated repression at the *P_coxB_* promoter required all three direct-repeat motifs (Figure 2B).

### The MHYT domain of CoxC is involved in gases recognition

**Figure 4.**
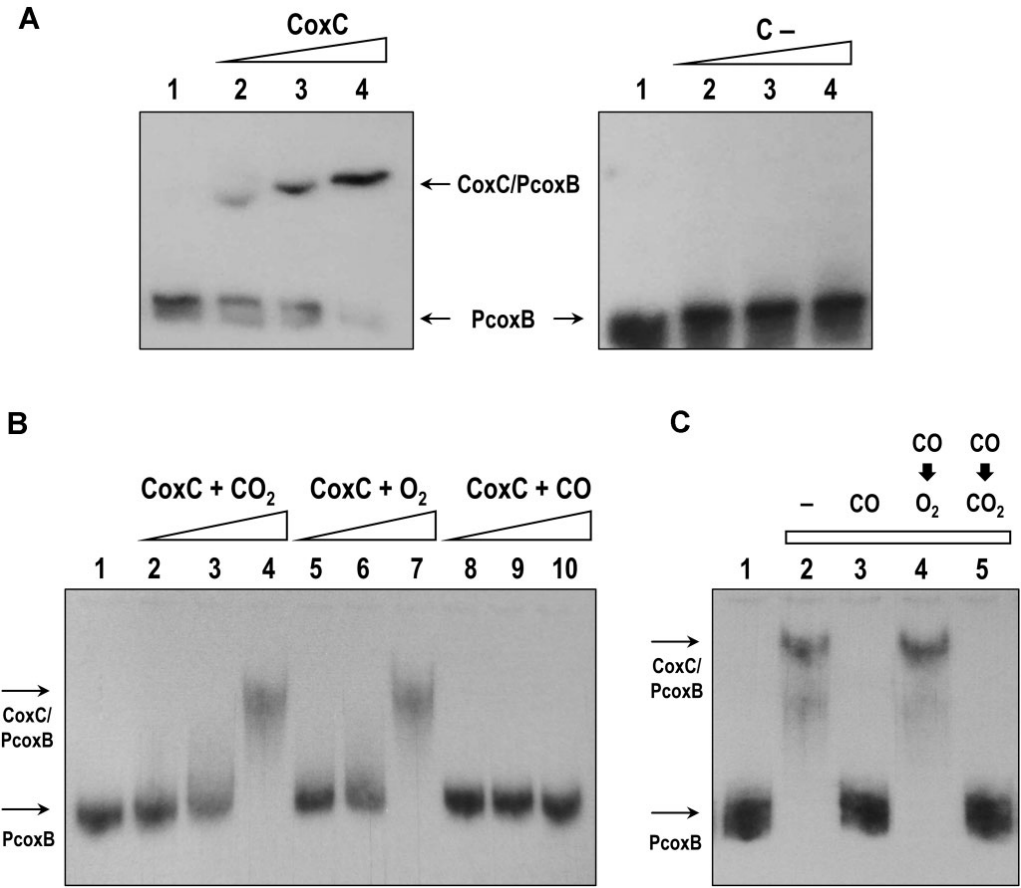
Interaction of membrane-bound CoxC protein with the *P_coxB_* promoter. EMSA are shown where the free PcoxB probe (lanes 1) and the CoxC/PcoxB complex are indicated by arrows. **(A)** Lanes 2–4 show EMSA of PcoxB with increasing concentrations, i.e. 0.1, 0.2, 0.5 mg/ml total protein, respectively, of the CoxC-enriched membrane fraction from *E. coli* DH10B (pIZ2-CoxC) cells expressing CoxC (CoxC) or with the membrane fraction from *E. coli* DH10B (pIZ2) cells serving as control (C–). **(B)** EMSA of PcoxB with increasing concentrations, i.e. 0.1, 0.2, 0.5 mg/ml total protein, of the CoxC-enriched membrane fraction treated with CO_2_ (lanes 2–4), O_2_ (lanes 5–7), or CO (lanes 8–10). **(C)** EMSA of PcoxB with 0.5 mg/ml total protein of the CoxC-enriched membrane fraction treated (lane 3), or untreated (lane 2) with CO. Lanes 4 and 5 show EMSA performed with the CoxC-enriched membrane fraction treated with CO, followed by treatment with O_2_ (lane 4) or CO_2_ (lane 5) to displace CO.

Above, we demonstrated that the N-terminal MHYT domain of CoxC is essential for the CO-dependent de-repression of the *P_coxB_* promoter in *in vivo* assays (Figure 2A), and established the function of the LytTR domain of CoxC for its binding to the *P_coxB_* promoter (Figure 3). Consequently, these findings strongly suggest the involvement of the MHYT domain of CoxC in CO recognition, potentially leading to the subsequent release of CoxC from the *P_coxB_* promoter. However, to corroborate this hypothesis and discard any potential indirect effect of CO (e.g. through another sensor protein present in the host cell), we conducted *in vitro* assays with the full-length CoxC protein. Given that MHYT domain-containing proteins are membrane-anchored (5,7), we utilized an enriched membrane fraction of *E. coli* DH10B (pIZ2-CoxC) cells as the source of membrane-bound CoxC regulatory protein. Consistent with our observations with the soluble C-CoxC protein, the membrane-bound native CoxC protein effectively retarded the migration of the PcoxB probe in a concentration-dependent manner (Figure 4A). In contrast, the membrane fraction of an *E. coli* DH10B strain harboring the empty pIZ2 plasmid (control sample) did not retard the PcoxB probe (Figure 4A), confirming that the membrane-bound CoxC protein was responsible for the observed retardation of PcoxB. Remarkably, binding of CoxC to the PcoxB probe was disrupted when the membrane-bound regulator was exposed to CO (Figure 4B, lanes 8–10), strongly suggesting that CO binds to CoxC and inhibits its interaction with *P_coxB_*. Notably, this CO-dependent effect on PcoxB binding was absent when using the C-CoxC truncated protein (Figure 3A, lane 10), leading to the conclusion that CO interacts with CoxC through its membrane-bound MHYT domain.

We examined other gases, such as O_2_ and CO_2_, as potential effectors of the MHYT domain of CoxC. In contrast to the observed effect with CO, neither O_2_ nor CO_2_ inhibited the binding of CoxC to the PcoxB probe (Figure 4B), consistent with the CO-dependent de-repression of *P_coxB_* observed *in vivo* (Figure 2A). However, these results did not rule out the possibility that O_2_ or CO_2_ may interact with the MHYT domain of CoxC without inducing the necessary conformational change to prevent interaction with the target promoter. To confirm this, we performed EMSA with full-length CoxC protein treated with CO, followed by displacement of CO by O_2_ or CO_2_. Figure 4C illustrates that while CO_2_ failed to prevent the CO induction effect (i.e. the PcoxB probe remained unbound), O_2_ successfully competed with CO, allowing the formation of the CoxC/PcoxB complex. Therefore, these findings demonstrate the reversible CO-dependent effect on CoxC and suggest that the MHYT domain can interact with both CO and O_2_, with these gases acting as agonist and antagonist, respectively.

### Copper is required for the interaction of CoxC with CO

**Figure 5.**
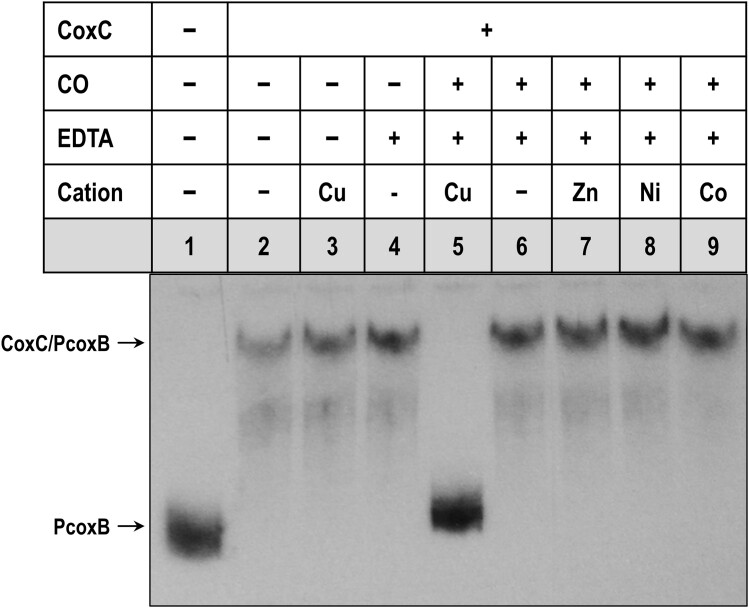
Effect of EDTA and various cations on the interaction between CoxC and the *P_coxB_* promoter. EMSA of PcoxB probe with the CoxC-enriched membrane fraction from *E. coli* DH10B cells containing the pIZ2-CoxC plasmid (0.5 mg/ml total protein). Lane 1, shows the free PcoxB probe; lanes 2–9 show EMSA with the CoxC-enriched membrane fraction treated (+) or untreated (–) with CO, 0.2 mM EDTA, 0.2 mM CuCl_2_ (Cu), 2 mM ZnCl_2_ (Zn), 2 mM NiCl_2_ (Ni) or 2 mM CoCl_2_ (Co). The free PcoxB probe and the CoxC/PcoxB complex are indicated by arrows.

It has been proposed that the conserved Met, His, and Tyr residues in MHYT domains coordinate copper ions, potentially enabling the sensing of diatomic gases such as O_2_, CO or NO (2). To determine whether divalent cations are indeed crucial for the CO-dependent conformational change that inhibits the binding of CoxC to *P_coxB_*, EMSA experiments were conducted with membrane-bound CoxC protein treated with the chelating agent EDTA. Initially, the CoxC-enriched membrane fraction was treated with EDTA or divalent cations, such as CuCl_2_, in the absence of CO to confirm that these compounds did not impact the formation of the CoxC/PcoxB complex (Figure 5, lanes 3–4).

When CoxC was treated with CO in the presence of EDTA, this chelating agent counteracted the CO-dependent effect, and a CoxC/PcoxB retardation band was observed (Figure 5, lane 6). Thus, these results suggest that divalent cations are necessary for the interaction of CoxC with CO. To assess the metal-binding specificity of CoxC, EMSA was performed with membrane-bound CoxC incubated in the presence of EDTA, CO, and four different divalent cations, i.e. Cu^2+^, Zn^2+^, Ni^2+^ or Co^2+^. Only in the presence of Cu^2+^ was CO able to disrupt the interaction between CoxC and the PcoxB probe (Figure 5, lanes 5 and 7–9). Therefore, all these findings demonstrate that CoxC requires Cu^2+^ to recognize CO as its effector molecule.

## Discussion

MHYT are transmembrane protein domains predicted to serve as sensor structures that likely interact with diatomic gases such as NO, CO and O_2_ (2,4,8). Despite their presence in many phylogenetically distant microorganisms, only a few proteins containing MHYT domains have been studied so far, all of which contain GGDEF-EAL motifs involved in c-di-GMP metabolism. Among these, MucR and NbdA proteins from *P. aeruginosa*, which belong to the group of MHYT domains fused to GGDEF-EAL motifs, play roles in NO-dependent biofilm dispersion. Notably, deletion of the MHYT domains in these proteins results in loss of their ability to induce biofilm dispersion (4). Furthermore, substitution of the conserved His residue with an Ala residue at the second MHYT motif of MucR abolishes nitrate-induced suppression of alginate production, suggesting the involvement of this MHYT motif in the nitrate response, likely through NO sensing (5). The MHYT domain is also responsible for the localization of CdgB, a protein with MHYT-PAS-GGDEF-EAL domain organization and bifunctional c-di-GMP cyclase/phosphodiesterase activity, to the membrane of *A. baldaniorum* Sp245 (7). However, despite the discovery of MHYT domains and the suggestion of their gas-sensory function, this assumption had not been experimentally demonstrated until now. In this study, we demonstrate that the CoxC protein from *A. carboxidovorans* OM5 is the first MHYT domain-containing protein reported to act as a transcriptional regulator, and we show that the MHYT domain confers upon this regulator the ability to sense CO as a gas effector molecule.

Through both *in vivo* and *in vitro* approaches, namely β-galactosidase assays conducted in *E. coli* cells containing the *P_coxB_::lacZ* reporter fusion and the *coxC* gene in *trans* (Figure 2A), and EMSA of the PcoxB probe with CoxC-enriched membrane fractions (Figure 3), we have demonstrated that CoxC functions as a transcriptional repressor that binds to the *P_coxB_* promoter located at the 5′-end of the *cox* cluster. The role of CoxC as a transcriptional regulator aligns with the presence of a predicted LytTR DNA-binding domain in the C-terminal half of the protein (Figure 1C). Additionally, CoxC responds to the presence of CO, as evidenced by the ability of this gas to alleviate the CoxC-mediated repression (Figure 2A) and binding (Figure 4) to the *P_coxB_* promoter. Notably, the CO-dependent activation of *P_coxB_* does not occur when using the truncated C-CoxC protein lacking the N-terminal MHYT domain (Figure 2A), consistent with the observation that CO fails to prevent the formation of a retardation complex between C-CoxC and the PcoxB probe (Figure 3A, lane 10). These findings suggest that the N-terminal MHYT domain of CoxC is responsible for CO sensing, while the C-terminal LytTR domain is involved in binding to the target promoter.

Other gases, such as CO_2_ or O_2_, did not act as effector molecules of CoxC, as they did not alleviate CoxC-mediated repression of *P_coxB_* (Figure 2A) or prevent the formation of a CoxC/PcoxB complex in EMSA (Figure 4B). Importantly, if CoxC functions as a CO sensor under aerobic conditions, CO binding should be reversible in the presence of O_2_. Accordingly, we successfully restored the binding of CO-activated CoxC to the PcoxB probe when CO was replaced by O_2_, whereas this effect was not observed when using CO_2_ to attempt to displace CO from CO-activated CoxC (Figure 4C). Therefore, these results provide solid evidence that CoxC is capable of sensing CO concentration under aerobic conditions, which has physiological significance since the cox pathway is functional only in the presence of O_2_.

It has been proposed that the conserved Met, His, and Tyr residues in the MHYT domain could provide up to eight coordinating bonds, which are sufficient for the coordination of one or two copper atoms in the membrane (2,29). The results presented in this study confirm that copper, but not zinc, nickel, or cobalt, is the divalent cation required for the interaction of CO with CoxC (Figure 5), providing the first experimental confirmation of the previous hypothesis regarding the role of copper in the gas-sensing mechanism of MHYT domains (2). Although the precise molecular dynamic properties must await the availability of structural data on the CoxC protein, we hypothesize that when the CO concentration is high, this gas displaces O_2_ (which could be bound to CoxC under aerobic conditions) and triggers the necessary conformational change in CoxC to facilitate its release from the target promoter, thereby inducing gene expression.

**Figure 6.**
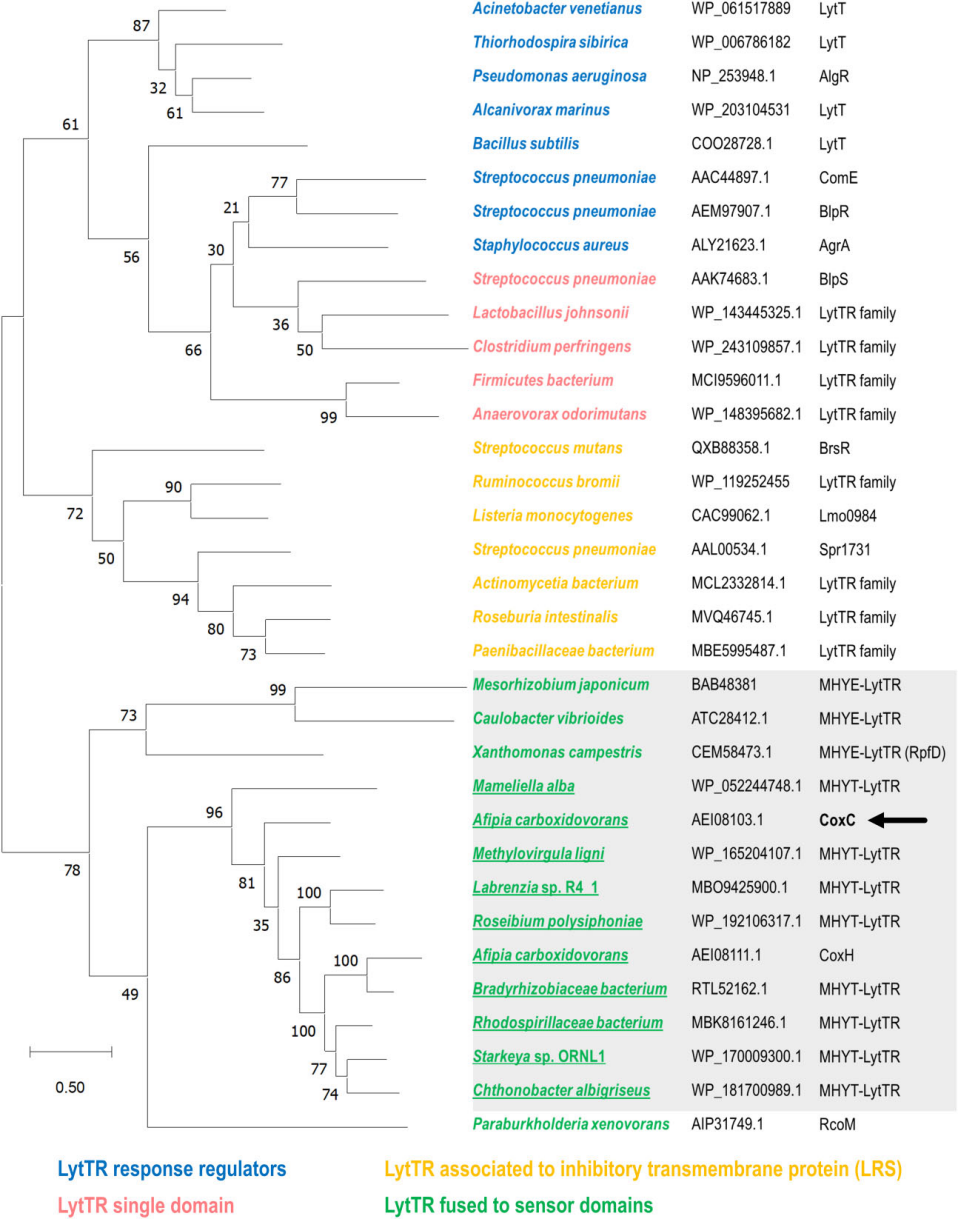
Phylogenetic tree of LytTR-type transcriptional regulators. Multiple sequence alignments of various proteins were performed using the Clustal W program. The maximum likelihood phylogenetic tree was constructed utilizing the neighbor-joining method, with numbers at the nodes representing the percentages of bootstrap values from 250 replicates (details provided in Materials and methods). The tree is drawn to scale, with branch lengths measured in the number of substitutions per site (as indicated by the black bar). The names of the bacteria, along with the corresponding GenBank accession numbers and names of the LytTR proteins analyzed, are shown. A color code is used to differentiate proteins belonging to different families of LytTR regulators. Membrane-bound LytTR regulators are shaded, and CoxC-like members are underlined. The CoxC protein from *A. carboxidovorans* is indicated with an arrow.

Atmospheric CO is generated by natural processes, such as heme breakdown, as well as industrial activities (30–33). Some microorganisms can utilize CO as an energy and/or carbon source under both anoxic and aerobic conditions, and recent studies have revealed that CO oxidation is widespread, enabling the survival of heterotrophic bacteria under carbon starvation conditions (34–36). In all these bacteria, the expression of genes encoding CO dehydrogenases is under the control of CO-dependent transcriptional regulation systems (28,37–39). To date, two distinct CO-specific sensor proteins, CooA from *Rhodospirillum rubrum* and RcoM1 from *P. xenovorans* LB400, have been described to regulate anaerobic and aerobic CO dehydrogenase encoding genes, respectively (26–28,31,40). These proteins act as single-component transcriptional regulators composed of an N-terminal CO-binding heme sensor domain that allosterically activates a C-terminal DNA-binding effector domain. While the DNA-binding domain of CooA contains the well-studied helix-turn-helix (HTH) motif and belongs to the CRP/FNR structural superfamily, RcoM1 contains a C-terminal LytTR DNA-binding effector domain (27,28,37). The LytTR fold exhibits a distinct DNA binding mode from that of the well-studied HTH motif, utilizing a conserved 10-stranded β-fold to mediate DNA binding via three elongated β-sheets (8,41–43). LytTR domains are typically found in various virulence-associated response regulators in pathogenic bacteria, although some are single-domain proteins or associated with an inhibitory transmembrane protein (LRS) (8,44). The canonical DNA-binding site for such proteins consists of a pair of 9 bp direct repeats spaced 10–13 bp apart (8,45). However, the RcoM1 promoter binding site differs from those of other LytTR-containing transcription factors, consisting of three 8-bp imperfect direct repeats of the sequence TTCNNGCA with 12–13 bp spacing regions (45). As indicated above, sequence comparison analysis revealed that CoxC also contains a C-terminal LytTR domain exhibiting amino acid sequence identity to that of RcoM. The LytTR domain of CoxC was shown to bind to the *P_coxB_* target promoter in a protein concentration-dependent manner (Figure 3A), requiring the presence of all three 8 bp direct repeats (TTNNNGCN) at the operator region for efficient binding (Figure 3C). However, unlike RcoM1 and most LytTR-type regulators that bind upstream of the -35 region of the target promoter and function as transcriptional activators, the CoxC operator region overlaps the predicted -35 box of the promoter (Figure 1B), consistent with the role of CoxC as a repressor of *P_coxB_*. Moreover, CoxC exhibits another unprecedented feature among LytTR regulators described so far, as it is membrane-associated through its N-terminal MHYT domain. In fact, a phylogenetic tree of different types of LytTR members revealed that CoxC serves as the prototype of a subfamily of LytTR regulators, where the DNA-binding domain is fused to a membrane-bound sensor domain (Figure 6). This new subfamily comprises CoxC orthologs encoded near *cox* genes in the genomes of various α-proteobacteria, suggesting their involvement in the regulation of CO oxidation genes, along with other putative regulators containing three predicted transmembrane segments, termed MHYE domains, the function of which remains unknown (Figure 6) (8).

The fact that CoxC is the only membrane-bound CO-sensing regulator described so far, acting as a transcriptional repressor, may be linked to its physiological role in controlling the expression of *cox* genes involved in the aerobic oxidation of CO in bacteria that rely on this gas as their sole carbon and energy source. These carboxidotrophic bacteria encounter high CO concentrations (up to 90% (v/v) in the gas phase), which could be toxic upon entry into the cell (30,34,46,47). Therefore, the localization of CoxC at the cell membrane enables the extracellular detection of CO, thereby accelerating the expression of the CO oxidation machinery before high concentrations of this gas can enter the cytoplasm and cause severe toxicity. On the other hand, a CO-dependent de-repression of *cox* genes may have been favored in evolution over CO-dependent transcriptional activation, potentially offering a quicker induction mechanism that circumvents the need for a direct interaction of the regulator with the RNA polymerase.

In summary, this work provides experimental evidence that the widespread MHYT domain recognizes CO as an effector and mediates the allosteric regulation of a LytTR DNA-binding domain in the single-component CoxC transcriptional regulator. Furthermore, this research paves the way for the future development of new CO-sensing genetic biosensors.

## Supplementary Material

gkae575_Supplemental_File

## Data Availability

All data needed to evaluate the conclusions in the paper are present in the paper and/or Supplementary Data.
