# Supplementary material for: Characterization of a MHYT domain-coupled transcriptional regulator that responds to carbon monoxide
Source: Nucleic Acids Res. 2024 Jul 5;52(15):8849–60. doi: 10.1093/nar/gkae575 (PMC11347149; doi:10.1093/nar/gkae575)
Supplement: gkae575_Supplemental_File [file gkae575_supplemental_file.pdf]

Supplementary material

Characterization of a MHYT domain-coupled transcriptional regulator that responds to carbon monoxide

Gonzalo Durante-Rodríguez*, Sofía de Francisco-Polanco, José Luis García and Eduardo Díaz*

Department of Microbial and Plant Biotechnology, Centro de Investigaciones Biológicas Margarita Salas-CSIC. Calle Ramiro de Maeztu, 9. 28040. Madrid. Spain.

*To whom correspondence should be addressed. Tel. +34 918373112. Email: ediaz@cib.csic.es

Correspondence may also be addressed to Gonzalo Durante-Rodríguez. Tel. +34 918373112. Email: gdurante@cib.csic.es

Table S1. Bacterial strains and plasmids used in this study.

Strain or plasmid	Relevant genotype and characteristic(s)	Reference
**Bacterial strains**		
A. carboxidovorans OM5	Wild-type, CO oxidizing strain, harbours pHCG3 plasmid (*cox* genes)	(9)
E. coli DH10B	F´, *mcrA* Δ(*mrr hsdRMS-mcrBC*) φ80d*lac*ΔM15 Δ*lacX74 deoR recA1 araD139* Δ(*ara-leu*)*7697 galU galK* λ *rpsL endA1 nupG*	Invitrogen
E. coli M15	Strain for regulated high-level expression with pQE vectors	Qiagen
**Plasmids**		
pIZ2	Gm^r, *ori*pBBR1, Mob^+, *lacZα*, $P_{tac}/lacI^q$ derived from pIZ1016 with modified MCS	(48)
pIZ2-CoxC	Gm^r, pIZ2 derivative for expression of CoxC	This work
pIZ2-C-CoxC	Gm^r, pIZ2 derivative for expression of C-CoxC	This work
pQE32	Ap^r, *ori*ColE1, T5 promoter *lac* operator, λ t_0/*E. coli rrnB T1* terminators, N-terminal His_6	Qiagen
pQE32-His_6C-CoxC	Ap^r, pQE32 derivative for expression of His_6C-CoxC	This work
pREP4	Km^r, plasmid that expresses the *lacI* repressor	Qiagen
pSEVA225	Km^r, *ori*RK2, *lacZ* promoter probe broad host range vector	(49)
pSEVA225-PcoxB	Km^r, pSEVA225 derivative carrying the P_{coxB}::*lacZ* transcriptional fusion	This work
pSEVA225-PcoxB1	Km^r, pSEVA225 derivative carrying the P_{coxB1}::*lacZ* transcriptional fusion	This work

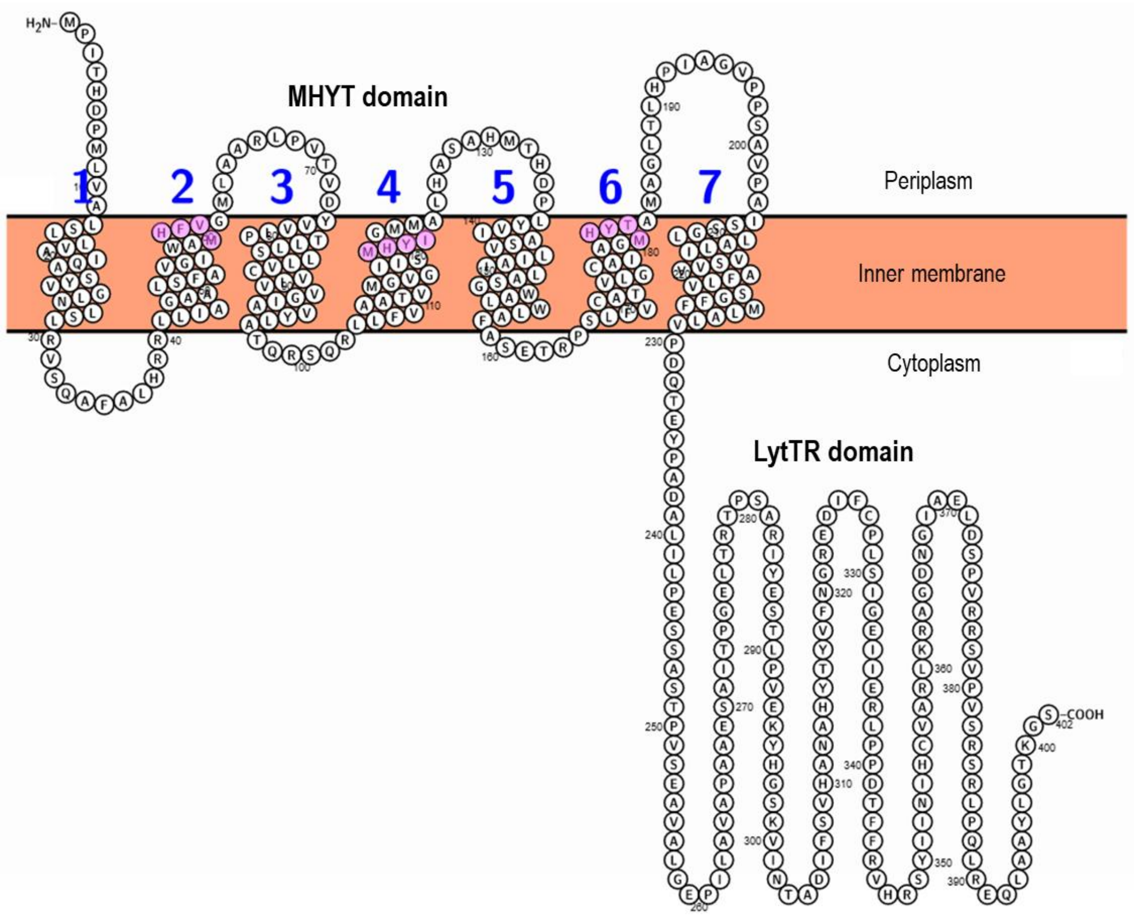

Figure S1. Model of the membrane topology of the CoxC protein. Circles represent the aa residues. The seven transmembrane regions are numbered and the MHYT motifs predicted to be involved in copper binding and sensing CO are shaded pink.

References

1. Acedos,M.G., de la Torre,I., Santos,V.E., García-Ochoa,F., García,J.L. and Galán,B. (2021) Modulating redox metabolism to improve isobutanol production in *Shimwellia blattae*. *Biotechnol. Biofuels*, **14**, 8.
2. Silva-Rocha,R., Martínez-García,E., Calles,B., Chavarría,M., Arce-Rodríguez,A., de Las Heras,A., Páez-Espino,A.D., Durante-Rodríguez,G., Kim,J., Nikel,P.I., *et al.* (2013) The Standard European Vector Architecture (SEVA): a coherent platform for the analysis and deployment of complex prokaryotic phenotypes. *Nucleic Acids Res.*, **41**, 666-675.
